# Medical and Philosophical Intelligence

**Published:** 1818-05

**Authors:** 


### MEDICAL AND PHILOSOPHICAL INTELLIGENCE.

### *Proceedings of Societies.*

ROYAL SOCIETY.—SIR EVERARD HOME has been making some microscopical experiments on the coagulation of blood, on certain tubes formed in coagula, and also in pus. To us they were not perfectly intelligible: we shall, therefore, suspend our opinion of them till they appear in print.

In the *Royal Society of Edinburgh*, Dr. MURRAY read the first part of a paper "On the Relation, in the Law of Definite Proportions in Chemical Combinations, to the Constitution of the Acids, Alkalies, and Earths, and their Compounds." Its object was to determine if the composition of these substances, according to the theory which he has lately proposed, be conformable to the law of definite proportions. The part of the paper read extended to the acids of which sulphur and carbon are the radicals, the vegetable acids being comprised under the latter. A very strict coincidence is found in the actual proportions, according to the theory, with the law, so as to afford proofs even of the truth of the former; and some of the results display views very different from those which have been hitherto proposed. The remainder of the paper will be read on a succeeding evening.

---

*Wernerian Society.*—In digging through a bed of alluvial clay, at Kilmarnock, to the depth of 17½ feet, four large tusks, resembling those of the elephant, were found; and also the rib of some large animal. The largest tusk measured 40 inches in length, and 12¼ inches in circumference at the maxillary extremity, and 8½ inches at the end. In the same bed of clay, remains of shells were found.

The Secretary read the first part of Dr. Traill's account of an African orang-outang, which lately died at Liverpool, the property of Mr. Bullock, of the Piccadilly Museum. In the introduction to his paper, he gave an account of the manners of the animal, as described by Captain Payne, who purchased it at Isle of Princes, from a native trader, who had brought it from the Gaboon river. Captain Payne had it in his custody for more than two months. It shewed an inclination to imitate many human actions; but never attempted the imitation of sounds. It disliked the erect posture, and walked on the knuckles, not the palms, of the fore extremities. It was dirty in its habits, and very timid. It associated familiarly with the crew, excepting one boy, to whom it shewed a decided and unceasing aversion. It was a faithful attendant of the seaman's mess; ate almost every kind of vegetable offered; was very fond of sweet articles of food, but did not relish any kind of butcher's meat. As the vessel approached the colder latitudes, it became somewhat languid, and carefully wrapped itself in a blanket on retiring to rest. From accounts given to Captain Payne by negro traders, on whose veracity he placed dependance, it appears that, in its native haunts, it is a very formidable animal: they all agreed, too, in affirming, that negro girls had sometimes been carried off, and kept in a state of frightful captivity for years; stories which have hitherto been regarded as resting wholly on the authority of Purchas's "Pilgrimes," and other old works.

Feb. 21.—The second part of Dr. Traill's paper on the African orang-outang was read, containing an account of the dissection of the animal. To this dissection both Dr. Traill and Dr. Vose, of Liverpool, dedicated all their leisure hours for several days. They found Tyson's descriptions, in many respects, accurate; but they also detected several mistakes and omissions. The appearances on dissection could scarcely be abridged: it may, however, be mentioned, that they found a flat triangular muscle inserted near the top of the thigh, which appears to have escaped the notice of Tyson, Camper, and Cuvier The action of this muscle is to draw the thigh up toward the body; and, from its being evidently calculated to facilitate climbing, it has been proposed to name it *Musculus scansorius*. The animal was a female; and the anatomists remark, that the peculiar form of the pelvis shows that the erect posture is not the natural one of the animal; for that, in the erect posture, it would be extremely liable to abortion in the gravid state.

Professor Jameson read some observations on the natural history of the diamond, and stated it as a conjecture, that the remarkable hardness of some woods may be owing to their containing carbonaceous matter, approaching to the adamantine state, and that the diamond itself might occur as a secretion in grain, or even in crystals, in some of the vegetables in the warmer regions of the earth. He also alluded particularly to the natural history of the tabasheer, or *vegetable opal*, found in some Oriental vegetables; and, from the great tendency observed in some vegetables to secrete silica, he offered, as a conjecture, that some silicified woods, met with on the surface of the earth, might be trunks or branches of trees, which had been killed by the over secretion of siliceous matter.

At the same meeting, the Secretary read a communication from Mr. Butter, surgeon of the South Devon Militia, giving an account of the change of plumage in the females of several gallinaceous birds, particularly common poultry, to that which resembles the males; and showing that this is a natural result of advanced age only, and not to be accounted a monstrosity.

At the meeting on Jan. 24, Mr. James Wilson communicated some remarks on the eggs of the common frog, and on the tadpole in its early state, tending to prove that the young animal derives its nourishment from the mass of gelatinous matter with which it is encompassed, and that by means of a filament, analogous to an umbilical cord, by which it is attached to the mass. Mr. Wilson also gave an account of the progressive development of the members or extremities of the animal, as observed by him.

---

Mr. Bartlett is chosen Sub-Librarian and Registrar to the *Medical Society*, in Bolt-court, in the room of Mr. Pettigrew, who has resigned. The Society have in contemplation to make further additions to their valuable library, by disposing of their triplicates, and of such works as, not being medical, are generally to be met with in other collections.

---

The following subjects, for which the Society for the Encouragement of Arts, Manufactures, and Commerce, has offered premiums, we conceive worth the attention of our readers, as connected with pathology in general:—

*Cure of the Rot in Sheep.*—To the person who shall discover to the Society the best and most effectual method of curing the rot in sheep, verified by repeated and satisfactory experiments; the gold metal.

It is expected that the candidates furnish accurate accounts of the symptoms and cure of the disease, together with the imputed cause thereof, and the actual or probable means of prevention, which, with proper certificates, must be delivered to the Society on or before the first Tuesday in February, 1818.

*Cure of the Foot-Rot in Sheep.*—To the person who shall discover to the Society the best and most effectual method of curing the foot-rot in sheep; the silver medal.

It is required that the cure be ascertained by repeated and satisfactory experiments, and the method of performing it be verified by proper certificates, delivered to the Society on or before the first Tuesday in February, 1818.

*Preventing the Ill Effects of Flies on Sheep.*—To the person who shall discover to the Society the most effectual means of protecting sheep from being disturbed and injured by flies; the silver medal.

It is required that the method be ascertained by repeated experiments, and that a certificate of its efficacy be delivered to the Society on or before the first Tuesday in February, 1818.

*Protecting Sheep.*—To the person who, in the year 1816, shall have protected the greatest number of sheep, not fewer than one hundred, by hovels, sheds, or any other means; the gold medal.

A particular account of the experiments made, with the advantages arising therefrom, together with the expense, and certificates of its utility, to be produced to the Society on or before the first Tuesday in March, 1818.

N. B. It is required that the certificates shall specify the length of time the sheep were so protected, and the manner in which they were maintained during that time; together with the general method of managing them.

*Feeding Cattle.*—For the best experiments on stall-feeding of cattle, (not less than five head,) to be continued for the space of twelve months, in order to prove the earliest maturity and greatest propensity to fatten, of the most approved breeds of cattle in Great Britain, or Ireland, specifying the nature of the food given, together with the daily consumption of each beast, with its weekly increase in weight, and such other observations as may be deemed of consequence; the gold medal.

A full account of the methods employed, and of the expenses attending the same, and certificates of the sundry matters stated, to be produced on or before the second Tuesday in January, 1818.

---

We have several times endeavoured to draw the attention of our readers to the subject of the Dry-Rot. It is become a most important question, and appears to us purely physiological. We suspect there are various kinds of it. The following is transcribed from the last number of the *Philosophical Magazine*:—

"The Eden sloop of war (new), which was lately sunk in Hamoaze, to endeavour to cure her of the dry-rot, has been risen, commissioned, and taken into dock. On opening her, she has been found defective in every part, and must undergo a thorough repair. The Topaze frigate, also ordered for commission, which was repaired not long since, is found to be in the same state. The Dartmouth frigate, built at Dartmouth, three years old, never at sea, is also undergoing a complete repair. Not a ship is taken into dock but is found to be nearly rotten. The very best ships do not average more than twelve years' existence. The San Domingo, 74, was ripped up (four years old) at Portsmouth. The Queen Charlotte, 110, was built at Woolwich, sent round to Plymouth, found rotten, and underwent a thorough repair: she was also several months under the care of Dr. Lukin, an Admiralty chemist, who received 5000*l.* for his ineffectual labours to stop the progress of vegetation in the ship. After a short cruise, the Queen Charlotte was laid up at Portsmouth, where she remains in a very defective state."

---

All the newspapers are full of accounts of the rot among the sheep, and some go so far as to say, that salt has proved a certain preventive. We, who see nothing of this valuable animal but in his passage to the slaughter-house, pretend not to form any accurate judgment on the subject: but, we take the liberty again to call on our brethren in the country for instruction. We feel particularly desirous that every one of our correspondents should, at least, ask the shepherds, farmers, graziers, and butchers, in their vicinity, what meaning they annex to the term, and by what symptoms they distinguish it. Our own enquiries only render the question more complicated. From some, we are informed, that the disease is distinguished by what are called *flucs*, an animal in shape resembling a flounder, and found in the liver: but we have seen these too often in well-fed healthy sheep, to be satisfied with this theory. Others impute it to *phthisis pulmonalis;* but this cannot be so highly infectious. Dr. Harrison considers it as the intermittent fever; and that all we can discover, is an enlarged and hardened spleen. All this induces us to suspect, that different diseases are, in different counties, called by one common name; or, that causes, in many respects similar, produce different effect from combinations not understood.

We have, also, a similar ambiguity to complain of, respecting what is usually called the *distemper* in dogs, and shall be equally thankful for any information.

---

*Water-Spout in the Isle of Wight.*—On Saturday, the 7th of March, a water-spout burst at Stenbury, near Whitwell, in the Isle of Wight, which did considerable injury. It was preceded by a violently agitated atmosphere, the noise of which, for half an hour, resembled a roar the most dismal and appalling. When the cloud poured forth its contents, it seemed to the inhabitants of

Stenbury farm as though the flood-gates of the sea had broken, and their destruction was inevitable: the water rolled down the hill in such irresistible torrents, that it beat down a lofty wall, flooded all the lower apartments of the farm, and set the cattle loose among the streams; the affrighted inhabitants seeking shelter, with their children, in the upper rooms. The terror and painful feelings are indescribable.

---

We could not find room in our last for the Report of the Royal Hospitals.

*Christ's Hospital.*

| | |
|---|---|
| Children put forth apprentices | 157 |
| Buried last year | 13 |
| Children now under care of the Hospital | 1196 |
| To be admitted on presentation | 130 |
| | 1496 |

*St. Bartholomew's Hospital.*

| | |
|---|---|
| Patients admitted, cured, and discharged last year— | |
| In-patients | 2599 |
| Out-patients | 5607 |
| Buried last year | 195 |
| Remaining under cure—In-patients | 350 |
| Out-patients | 300 |
| So that there have been under the care of this Hospital last year | 9051 |

*St. Thomas's Hospital.*

| | |
|---|---|
| Cured and discharged last year | 9165 |
| Remaining under cure—In-patients | 395 |
| Out-patients | 316 |
| Buried last year | 170 |
| | 10046 |

*Bethlem Hospital.*

| | |
|---|---|
| Remaining in the Hospital | 309 |
| Buried last year | 4 |
| Cured and discharged last year | 142 |
| Patients under cure | 196 |
| Ditto incurables | 65 |
| | 716 |

The most remarkable thing, in the above report, is the number of deaths in Christ's Hospital,—nearly one in a hundred, at an age considered generally the most favourable to human life.

Whilst we boast of charities in England, let us peruse the following; and, whilst we talk of penitentiaries, let us endeavour to copy Mr. Bennett's account of Ghent.

*Report made respecting the Hospitals in Paris, by a Society of Physicians, from materials furnished by M. le Marquis de Pastorat.*—[This report comprehends a period of ten years, from the 1st of January, 1804, to the 1st of January, 1814.

| | Individuals received. | | Proportion died. |
|---|---|---|---|
| Hotel Dieu..... | 101,595 | — | 1 in 4,93 |
| La Charité ..... | 27,857 | — | 1 — 7,08 |
| Enfants Malades | 20,667 | — | 1 — 4,75* |
| Venereal Hospital | | | |
| Men ........ | 13,638 | — | 1 — 56 |
| Women ...... | 12,163 | — | 1 — 67 |
| Children .... | 1,775 | — | 2 — 25 |
| Lying-in Hospital | 21,053 | — | 1 — 25 |
| Dead born ........................... | | | 1 — 22† |

In the latter hospital, 1155 midwives received instructions.

It was observed, that children, who at their birth did not weigh eight pounds, seldom lived.

A trial was made of suckling four children with goat's milk; but they all died in a short time.

Several trials were made to discover the best means to prevent the hardening of the cellular membrane of new-born infants. Sand-baths, vapour-baths, &c. produced no effect: the best means appeared to be to wrap the child in new fine oily wool, covering the wool with German taffety. A perspiration then takes place, which softens the hardened parts.

The number of children received into the hospital, in ten years, was 5921. The greatest year was 1812, when the number was 5394; the shortest year, 1804, when the number was 4159; the average, 4592;—male children, 2346; female, 2246.

The Salpetriere (for old women), in 1813, there were 4618; the average of deaths, 601 a year.

Bicetre (for old men); of 2405, the deaths were 420 a year.

As to mad people received in two houses, the number of men were 2154, and of women 2804. Of women's cases, 658 were owing to lying-in, menstruation, or milk in the breast; 166 to love;—with the males, there were only 37 from that latter cause.

It was observed, that, during the revolution, of those who went mad from political causes, the males were all wild aristocrats—the females all democrats.

---

* In the Hospital des Enfans Malades, the most part were only brought when at the last extremity.

† 193 had twins, and two women had three at a birth.

Of 2804 females received in the Salpetriere, 604 were cured the first year, 497 the second, and 148 afterwards. Of the same number, 382 died the first year, 227 the second, and 181 afterwards.

The total number of sick persons treated in the different hospitals in ten years was 355,662, of whom 47,861 died.

The number of old and infirm people was 59,032, of whom 12,577 died.

The average expense of a sick person per day was 1 fr. 65 cents, or 1s. 4d.; and that of the expense, from first to last, 66 fr. 30 cents. or 2*l.* 14s. In the hospitals, the daily expense of a patient was 90 cents. or 9d. sterling.

The number of persons who received aid in their own houses, in 1813, was 102,806.

---

Professor Berzelius has given an account of a new alkali and metal, which he calls Selenium: it was found in a mineral, in a mine near Uten, in Sweden.

---

Mr. William Newnham, Surgeon, Farnham, has in the press an Essay on the Symptoms, Causes, and Treatment, of Inversio Uteri, with a History of the successful Extirpation of that Organ during the chronic Stage of the Disease.

---

Speedily will be published, a new edition, considerably improved, of Dr. Withering's Systematic Arrangement of British Plants, with an easy Introduction to the Study of Botany, in 4 vols. 8vo. illustrated by copper-plates.

---

Dr. Adams's new edition of Hunter on the Venereal Disease, with additions, will appear in a few days.

---

Mr. Robert Mac William, architect, has in the press, An Essay on the Origin and Operation of the Dry-rot; in which the source of the disease is investigated, with a view to establish the modes of prevention and cure on rational principles. It will make a quarto volume, illustrated with plates; and to it will be annexed suggestions on the cultivation of forest trees, with abstracts of the forest laws from the earliest times.

[We sincerely wish medical gentlemen would acquaint themselves with the exact meaning of the term "dry-rot." Whether it is ascertained, as asserted by some, always to begin from the earth, and to be attended with a web? Whether it is contagious, as others assert? But, first of all, an exact description of the disease, if we may so call it, and, particularly, whether the boletus usually growing from such wood is the cause or effect of the dry-rot? Lastly, by analysis ascertain, whether the wood thus decayed contains volatile alkali, or any other properties more particularly considered as the product of animals.]

Mr. TAUNTON's Summer Course of Lectures, on Anatomy, Physiology, Pathology, and Surgery, will commence on Saturday, May 23, 1818, at eight o'clock in the evening; and be continued every Tuesday, Thursday, and Saturday, at the same hour.

---

### REPORT OF DISEASES.

THE preceding month has been extremely unfavourable to those subjects, described in our last, as threatened with phthisis pulmonalis. Most of the cases of pneumonia, which were not met with early bleeding, have ended in abscesses, which the succeeding cold weather has rendered truly formidable. Other complaints still continue inflammatory, but less acute; and the mild weather, with which the month concluded, has produced a very favourable change in diseases of every description: this is less to be wondered at, as, whatever might have been their cause, they usually partook of an inflammatory diathesis.

The extract of Belladona has proved almost magically successful in a great number of complaints in the head, whether resembling tic douloureux, or partaking of an intermitting form. They have been principally in females; and very small doses, one or two grains, repeated every two hours *durante dolore*, have been sufficient. Sometimes a permanent cure has been produced by a single exhibition. In none has it been required beyond a third, slight, and very short paroxysm. The patient has always been advertised of the probable consequences to her sight, and other faculties. These have been, in some, considerably affected; in others, not at all.

A case of long-continued serous discharge, *per vaginam*, in an aged female, has puzzled the reporter a great deal. By local bleeding, externally, the pain has been removed, but the discharge continues unabated and unaltered. Though apparently serous, it has the smell which usually attends all discharges from those parts. The general health and appetite are good.

Hooping-cough has been extremely prevalent, and proved fatal to several infants during the cold season. Others are becoming convalescent, and, in such, the present mild weather will probably produce a cure. We have heard of great success by a fixed graduated temperature, which is said to have shortened the disease considerably, and without any inconvenience from a subsequent exposure to the open air. Measles are disappearing; and we hear nothing of scarlatina. The alarm concerning typhus is, we believe, nearly subsided. By Mr. Field's Report of Christ Hospital, small-pox, in a mild form, has been frequent *post vaccinationem*. We hear the same from other quarters; but, happily, it induces no disadvantageous change in the public opinion relative to that valuable discovery.

### METEOROLOGICAL JOURNAL,

By Messrs. William Harris and Co. 50, Holborn, London.

*From the 20th March to the 19th April, inclusive.*

| Day of Month. | Moon. | Rain gauge | Therm. | | | Barom. | | De Luc's Hygrom. Dry | | Damp | | Wind. | | Atmospheric Variation. | | |
|---|---|---|---|---|---|---|---|---|---|---|---|---|---|---|---|---|
| Mar. | | | | | | | | | | | | | | | | |
| 20 | | | 50 | 55 | 38 | 29·84 | 29·91 | 0 | 0 | 0 | 0 | WNW | W | Small Rain | Fine | |
| 21 | | ,12 | 43 | 51 | 42 | 29·94 | 29·80 | | | 10 | 15 | W | SW | Fine | Rain | Fine |
| 22 | ○ | ,09 | 44 | 50 | 47 | 29·68 | 29·48 | | | 16 | 17 | WSW | SW va. | Rain | Fine | Rain |
| 23 | | ,21 | 48 | 51 | 45 | 29·31 | 29·65 | | | 19 | 17 | SW va. | SW | Rain | Showery | Clo. |
| 24 | | ,20 | 47 | 50 | 38 | 29·65 | 29·81 | | | 12 | 11 | SW | SW | Fine | Hail | Fine |
| 25 | | | 40 | 47 | 37 | 29·54 | 29·84 | | | 17 | 14 | SSW | SW | Clo. | Rain | Fine |
| 26 | | ,11 | 40 | 41 | 35 | 29·52 | 29·51 | | | 18 | 15 | SSE | NW | Rain | | Fine |
| 27 | | ,19 | 39 | 44 | 37 | 30·00 | 30·25 | | | 17 | 15 | NW | NNE | Fine | Showery | Fine |
| 28 | | ,13 | 41 | 46 | 39 | 30·30 | 30·26 | | | 10 | 9 | SSE | SE | Clo. | | |
| 29 | ◑ | ,05 | 43 | 52 | 39 | 30·16 | 30·10 | | | 11 | 7 | SE | SE | Fine | | |
| 30 | | ,03 | 45 | 52 | 39 | 30·14 | 30·26 | | | 5 | 10 | SSE | N | Fog | Fine | |
| 31 | | ,02 | 41 | 46 | 39 | 30·30 | 30·27 | | | 16 | 16 | N | N | Fine | | |
| Apr. | | | | | | | | | | | | | | | | |
| 1 | | | 42 | 47 | 39 | 30·21 | 30·20 | | | 17 | 15 | NE | NE | Fine | | |
| 2 | | | 40 | 46 | 40 | 30·26 | 30·29 | | | 17 | 15 | NNE | NNE | Clo. | Fine | |
| 3 | | | 41 | 46 | 40 | 30·34 | 30·37 | | | 12 | 14 | NNE | NNE | Fine | | |
| 4 | | | 42 | 44 | 39 | 30·30 | 30·10 | | | 11 | 12 | E | E | Fine | | |
| 5 | ● | | 42 | 47 | 40 | 30.04 | 29·66 | | | 9 | 7 | SSE | S | Fine | | |
| 6 | | | 42 | 43 | 40 | 29·65 | 29·60 | | | 10 | 12 | W va. | S | Rain | Clo. | Fine |
| 7 | | ,11 | 40 | 45 | 49 | 29·74 | 29·48 | | | 17 | 25 | SE | SSE | Rain | | |
| 8 | | ,09 | 54 | 55 | 50 | 29·48 | 29·38 | | | 20 | 18 | SSW | S | Fine | Rain | |
| 9 | | ,13 | 55 | 55 | 51 | 29·41 | 29·52 | | | 17 | 12 | SSW | SSW | Rain | Fine | |
| 10 | | ,07 | 55 | 55 | 50 | 29·35 | 29·31 | | | 10 | 9 | SW | SW | Rain | | Fine |
| 11 | | ,05 | 53 | 55 | 38 | 29·41 | 29·68 | | | 15 | 11 | W | WNW | Fine | Rain | |
| 12 | | | 40 | 47 | 39 | 29·77 | 30·08 | | | 11 | 9 | W | W | Fine | Rain | Fine |
| 13 | ◐ | ,14 | 43 | 49 | 42 | 30·03 | 29·83 | | | 5 | 9 | SW | SE | Fine | | |
| 14 | | | 47 | 51 | 41 | 29·57 | 29·75 | | | 12 | 12 | SE | SSE | Fine | | |
| 15 | | | 44 | 49 | 47 | 29·60 | 29·58 | | | 9 | 13 | SE | ENE | Fine | | |
| 16 | | | 50 | 55 | 41 | 29·37 | 29·32 | | | 5 | 13 | E | E | Fine | | Rain |
| 17 | | ,10 | 45 | 52 | 44 | 29·31 | 29·29 | | | 12 | 15 | ENE | ENE | Rain | Fine | |
| 18 | | | 49 | 53 | 37 | 30·12 | 30·22 | | | 7 | 12 | ENE | ENE | Clo. | Fine | |
| 19 | | ,05 | 41 | 47 | | 29·71 | 29·82 | | | 9 | 10 | NNE | E | Fine | | |

